# A Sensitive Method for Detecting Peptide-specific CD4^+^ T Cell Responses in Peripheral Blood from Patients with Myasthenia Gravis

**DOI:** 10.3389/fimmu.2017.01370

**Published:** 2017-10-24

**Authors:** Sapna Sharma, Clas Malmeström, Christopher Lindberg, Sarah Meisel, Karin Schön, Martina Verolin, Nils Yngve Lycke

**Affiliations:** ^1^Department of Microbiology and Immunology, Institute of Biomedicine, University of Gothenburg, Gothenburg, Sweden; ^2^Laboratory for Clinical Immunology, Sahlgrenska University Hospital, Gothenburg, Sweden; ^3^Department of Neurology, Sahlgrenska University Hospital, Gothenburg, Sweden; ^4^Toleranzia AB, Gothenburg, Sweden

**Keywords:** myasthenia gravis, acetylcholine receptor, autoimmune disease, dendritic cells, CD4^+^ T cells, peripheral blood and peptide

## Abstract

Myasthenia gravis (MG) is an autoimmune neurological disorder typified by skeletal muscle fatigue and most often production of autoantibodies against the nicotinic acetylcholine receptor (AChR). The present study was undertaken to assess the extent of AChR-peptide recognition in MG patients using co-culturing (DC:TC) of autologous monocyte-derived dendritic cells (moDCs) and highly enriched CD4^+^ T cells from the blood as compared to the traditional whole peripheral blood mononuclear cell (PBMC) cultures. We found that the DC:TC cultures were highly superior to the PBMC cultures for detection of reactivity toward HLA-DQ/DR-restricted AChR-peptides. In fact, whereas DC:TC cultures identified recognition in all MG patients the PBMC cultures failed to detect responsiveness in around 40% of the patients. Furthermore, reactivity to multiple peptides was evident in DC:TC cultures, while PBMC cultures mostly exhibited reactivity to a single peptide. No healthy control (HC) CD4^+^ T cells responded to the peptides in either culture system. Interestingly, whereas spontaneous production of IFNγ and IL-17 was observed in the DC:TC cultures from MG patients, recall responses to peptides enhanced IL-10 production in 9/13 MG patients, while little increase in IFNγ and IL-17 was seen. HCs did not produce cytokines to peptide stimulations. We conclude that the DC: TC culture system is significantly more sensitive and better identifies the extent of responsiveness in MG patients to AChR-peptides than traditional PBMC cultures.

## Introduction

Myasthenia gravis (MG) is an organ-specific autoimmune disease with a reported prevalence of 14–17 cases/100,000 in a Swedish/European or US population (1, 2). It is an antibody-mediated, CD4^+^ T cell-dependent neuro-inflammatory disorder, which results in loss of function in the acetylcholine receptor (AChR) at the neuromuscular junction (3, 4). This leads to a long-standing, debilitating disease with clinically diverse manifestations and severity. Roughly 85% of MG patients have autoantibodies that bind to the nicotinic AChR, which consists of a transmembrane glycoprotein composed of five subunits with a stoichiometry of α_2_βγδ. Among the four different subunits of AChR, it is the α-chain that is the prime target for an autoimmune attack in MG (5, 6). There are at least three different forms of AChR autoantibody-associated MG, indicating that the etiology may be diverse and, hence, MG constitutes a heterogenic patient group (7, 8). In the first group, early onset of MG (EOMG) more females than males are affected, while in the second group, late onset of MG (LOMG) disease is more prevalent in males (2, 9, 10). In both groups, the disease onset is often gradual with fluctuating muscle fatigability and weakness, which typically aggravate during the initial two years. A third group is MG patients presenting with thymoma (TOMG) (7). Beside these three groups, there is also a small group of patients who lack AChR antibodies but have antibodies against the muscle-specific receptor tyrosine kinase (MuSK MG). However, this group is also likely to be MG with a different etiology (8).

There is a genetic risk of developing MG and siblings or first grade relatives have a 4.5% increased risk of MG (7). Several studies have documented a strong association between MG and several HLA alleles (1, 11). Patients with EOMG have a distinct HLA association with HLA-DR3/DQA1/DRB1 as revealed in genome-wide association studies (GWAS) (12, 13). Furthermore, Maniaol et al. reported on the susceptibility to acquire MG disease in a Caucasian population and identified DRB1*15:01 as a major risk allele for LOMG and DRB1*03:01 as a risk factor for EOMG, indicating etiological differences between the two (14). Also, the DQB1* 05:02 locus has been associated with MG (15). Overall HLA-DR3, DQ8 and DQ6-restricted CD4^+^ T cell recognition of the AChR appear to be the most frequent in MG patients when consulting the immune epitope database (IEDB) (16). Apart from an association of MG with HLA class II regions, associations with class I regions have also been identified, specifically to HLA-B*08 (17). Of note, there is usually no association with HLA class II regions in MG patients with thymoma (14). GWAS studies have also identified a link to the locus encoding the CTLA4 protein, which is associated with regulatory CD4^+^ T cells (Tregs) (18, 19). Indeed, it is generally thought that an insufficient Treg function could be involved in the etiology of MG (20, 21). Studies using the experimental autoimmune myasthenia gravis (EAMG) model in mice or rats have clearly identified a protective role of Tregs against disease (22).

The formation of high-affinity anti-AChR autoantibodies requires CD4^+^ T cells that recognize the AChR (23). Therefore, T cell recognition of the AChR has been studied extensively and many publications have identified peptides from the extracellular α-chain, in particular, as the main targets (16). Importantly, recognition of epitopes persists over time in MG patients (23, 24). Hence, in theory, it appears feasible to develop treatment protocols for tolerization of autoreactive CD4^+^ T cells by using immunodominant epitopes from the AChR (25). Indeed, several studies have documented effective suppression of auto aggressive CD4^+^ T cell-mediated disease in EAMG models using defined peptides (26–30). Clinical studies in MG patients using this approach still await to be undertaken (31, 32). The amino acid sequences used for these experimental peptide-based therapies were derived from the many studies of peptide-specific T cell reactivity in MG patients. However, the frequency and unique peptide reactivity patterns have varied considerably in the different reports. It also remains a question as to the existence of a hierarchical order of dominant to subdominant AChR-peptide epitopes in MG patients (33). Whereas all previous studies have used isolated whole peripheral blood mononuclear cells (PBMCs) for culturing with the different AChR-peptides, no study has yet, to our knowledge, attempted to culture highly enriched CD4^+^ T cells with autologous dendritic cells (DC) (34). Such an approach could further advance our knowledge about the actual frequency and hierarchical dominance of CD4^+^ T cell reactivity to AChR-peptides in MG patients.

The present study was undertaken to investigate whether a more sensitive method compared to culture of whole PBMCs for detection MG-peptide-specific CD4^+^ T cells could be established. To this end, we adopted a method in which highly enriched CD4^+^ T cells from PBMCs were frozen and 5 days later thawed and co-cultured together with autologous monocyte-derived DCs (moDCs) and a selected panel of peptides from human AChR (35).

## Materials and Methods

### MG Patient Samples

The study was approved by the regional ethical review board at the University of Gothenburg, Sweden. Thirteen patients (age 28–86 years, and mean ± SD 56.8 ± 20 years) were included in the study and blood samples were obtained after written informed consent. There were five cases with an early onset (<50 years; mean onset age 36 ± 8 years) of disease and eight cases with a late onset of disease (>50 years; mean 69 ± 13 years) with the clinical diagnosis of MG (Table 1). For comparisons, samples from 10 age-matched blood donors with no history of disease were used as healthy controls (HC). Diagnosis among MG patients was made by an experienced neurologist (Christopher Lindberg) based on diagnostic criteria for MG consisting of the presence of muscle weakness and fatigability, a clinical condition responsive to anti-cholinesterase medication, and, if needed, neurophysiological assessments. Anti-AChR antibodies were detected in sera from most patients as indicated (Table 1).

**Table 1:** A compilation of clinical parameters for Myasthenia gravis (MG) patients enrolled in the present study.

S. No.	Age	Gender	New diagnosis (y/n)	AChR Abs (>0.45)	MUSK Abs	HLA		
MG-1	28	f	y	0.64	n	DRB1*03 (DR17), *04	DQA1*03, *05:01	DQB1 *02, *03 (DQ7)
MG-2	33	f	n	>20	n	DRB1*04, *13	DQA1*01, *03	DQB1 *03 (DQ8), *06
MG-3	33	f	n	11	n	DRB1*04, *14:04	DQA1*01, *03	DQB1 *03 (DQ7), *05
MG-4	36	f	n	>20	n	DRB1*08, *15	DQA1*01, *04:01	DQB1 *04, *06
MG-5	50	f	n	1.9	n	DRB1*03 (DR17), *04	DQA1*03:01, *05:01	DQB1 *02, *03 (DQ8)
MG-6	52	f	n	>20	n	DRB1*03 (DR17), *04	DQA1*03:01, *05:01	DQB1 *02, *03 (DQ8)
MG-7	55	m	n	>20	n	DRB1*15	DQA1*01	DQB1 *06
MG-8	60	f	n	n	n	DRB1*01, *11:04	DQA1*01, *05	DQB1 *03 (DQ7), *05
MG-9	64	m	y	>20	n	DRB1*10, *15	DQA1*01	DQB1 *05, *06
MG-10	74	f	n	n	n	DRB1*13, *14	DQA1*01	DQB1 *05, *06
MG-11	82	f	n	3.6	n	DRB1*11, *15	DQA1*01, *05	DQB1 *03 (DQ7), *06
MG-12	86	f	y	45	n	DRB1*04, *13	DQA1*01, *03	DQB1 *03 (DQ8), *06
MG-13	86	m	n	>20	n	DRB1*04, *15	DQA1*01, *03	DQB1 *03 (DQ7), *06

*Patients were selected on the basis of a clinical diagnosis of MG and not being on immunosuppressive treatments. f, female; m, male; y, yes; n, no; hAChR Abs, human acetylcholine receptor-specific antibodies in serum (n is negative); MUSK Abs, muscle-specific kinase (n is negative); HLA typing, human leukocyte antigen*.

### HLA Haplotype Determinations

The HLA-typing was performed with LABType^®^, Luminex xMAP technology, reverse SSO DNA typing method (One Lambda, USA), according to the standard operating procedure for tissue typing used at the Sahlgrenska University Hospital, Gothenburg, Sweden.

### Peptides

The six overlapping peptides representing the extracellular part of the α-chain of hAChR (residues α1–210) were produced and purified by Pepscan Technology (Netherlands). Six selected peptides covering amino acid 1–210 of the α-chain of hAChR (P1, P2, P3, P4, P5, and P6), identified in previous publications, were used in the present study (Table 2) (13, 36). A mixed peptide pool, PM-CEFT-MHC-II (PepMix^TM^), representing multiple antigens (JPT Peptide Technology, Germany), was used as a positive control. The mixed peptide pool consisted of 14 peptides each corresponding to defined HLA class II restricted T-cell epitopes (from *Cytomegalovirus, Epstein Barr virus, Influenza virus*, and *Clostridium tetani*) that were selected on the basis of a high frequency of responsiveness of PBL in healthy volunteers.

**Table 2:** Amino acid sequences of selected peptides P1–P6, representing the extracellular part of the hAChR receptor (residues α1–210).

Peptide	Sequence position	Amino Acid Sequence
P1	12–49	H-FKDYSSVVRPVEDHRQVVEVTVGLQLIQLINVDEVNQI-OH (38 aa)
P2	48–67	H-LGTWTYDGSVVAINPES-OH (20 aa)
P3	75–115	H-VKKIHIPSEKIWRPDLVLYNNADGDFAIVKFTKVLLQYTGH-OH (41 aa)
P4	78–93	H-IHIPSEKIWRPDLVLY-OH (15 aa)
P5	146–162	H-LGTWTYDGVVAINPES-OH (17 aa)
P6	195–212	H- DPTYLDITYHFVMQRLPL-OH (18 aa)

### Preparation of PBMCs

Peripheral blood mononuclear cells were isolated from 50 ml of freshly drawn blood using standard Ficoll-hypaque (density of 1.077 ± 0.001 g/ml) density gradient centrifugation (37). Briefly, heparin-treated fresh blood was diluted in PBS of equal volume. Then, 9 ml of Ficoll was added slowly to a 50-ml centrifuge tube followed by addition of 15 ml of diluted blood. Centrifugation was carried out at 22°C for 20 min at 250 x *g*. The mononuclear cells at the interface were collected and washed twice in PBS. These cells were used for differentiation of moDCs and for enrichment of CD4^+^ T cells.

### Generation of Monocyte-Derived Dendritic Cells

Isolated PBMCs were subjected to AutoMacs magnetic separation (Miltenyi Biotech) to deplete T and B cells. This process involved incubation with anti-human CD3^+^ (Cat no. 130-050-101, Miltenyi Biotech) and CD19^+^ microbeads (Cat no. 130-050-301, Miltenyi Biotech). The depleted PBMC were adjusted to 5 × 10^6^ cells/ml in incomplete Iscove’s medium (IMDM) (Biochrom, Berlin, Germany) containing 1 mmol/l l-glutamine and 50 µg/mL gentamicin (Sigma-Aldrich). The cell suspension was added to 6-well plates (Nunc) and incubated at 37°C in an atmosphere with 5% CO_2_ for 4 h. Cells that remained in suspension were removed, while adherent monocytes were maintained in 10% heat inactivated fetal bovine serum (FBS) supplemented IMDM medium and stimulated with 1,000 U/ml rhGM-CSF and 1,000 U/ml rhIL-4. On Day 6, immature DCs were collected and seeded into a 96-well plate at a cell density of 1 × 10^5^/ml (10^4^/well).

### CD4^+^ T Cell Enrichment

Negative selection of CD4^+^ cells was performed using a biotin-conjugated antibody cocktail (Cat no. 130-096-533, Miltenyi Biotech) specific for the lineage antigens CD8, CD14, CD15, CD16, CD19, CD36, CD56, CD123, TCR γ/δ, and CD235a on an AutoMACS (Miltenyi Biotech) for cell separation. Negative fractions were collected, washed, and counted using a hemocytometer. CD4^+^ T cells were stored in liquid N_2_ to be used later in the DC:TC co-culture experiments.

### Flow Cytometry

To examine the expression of surface markers on moDC (1 × 10^6^ cells/ml) the cells were incubated with primary antibodies on ice for 30 min. Antibodies used for flow cytometry included HLA-DRperCP, CD11cPE, CD14PEcy7 (BD Biosciences, USA). Data were acquired by flow cytometry on a LSR II instrument (BD biosciences) and analyzed using FlowJo (Tree star, USA).

### Optimization of DC:TC Coculture Conditions

**Figure 1:**
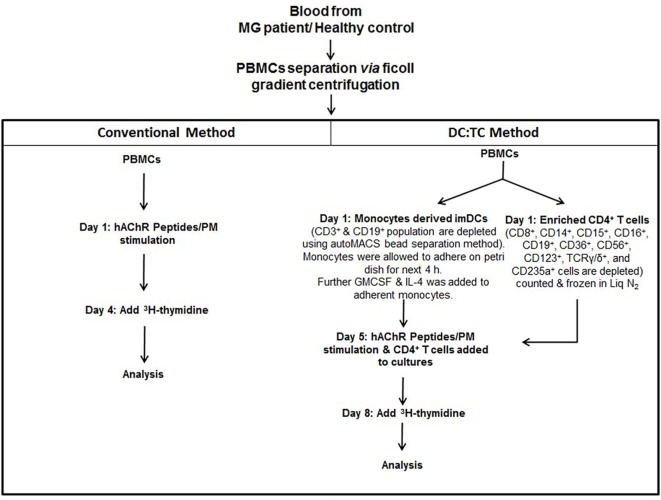
Flow Chart: This flow chart depicts two different cell culture strategies, conventional PBMC and DC:TC co-cultures, for detecting acetylcholine receptor (AChR)-peptide-specific CD4^+^ T cell responses in MG patients. Briefly, PBMCs were separated using standard Ficoll gradient centrifugation. Following washing in PBS whole PBMCs were stimulated with indicated peptides in 96-well plates at 2 × 10^5^ mononuclear cells/well and cell proliferation was measured after 72 h by 3 H^3^-thymidine incorporation. The DC:TC cultures relied on an initial pre-culture of enriched monocytes at 1 × 10^5^ cells/well in medium with GM-CSF and IL-4 to generate moDCs. After 5 days enriched syngeneic CD4^+^ T cells were thawed (stored in −70°C) and added to the mDC cultures at 1 × 10^6^ CD4^+^ T cells/well and stimulated by selected peptides, as indicated. Proliferation was assessed after 72 h in the co-cultures by addition of 3 H^3^-thymidine. Supernatants were harvested for determinations of cytokine production. Abbreviations: PBMCs, peripheral blood mononuclear cells; MG, myasthenia gravis; imDC, immature Dendritic cells; TC, T cell; hAChR, human acetylcholine receptor; PM, pepmix.

**Figure 2:**
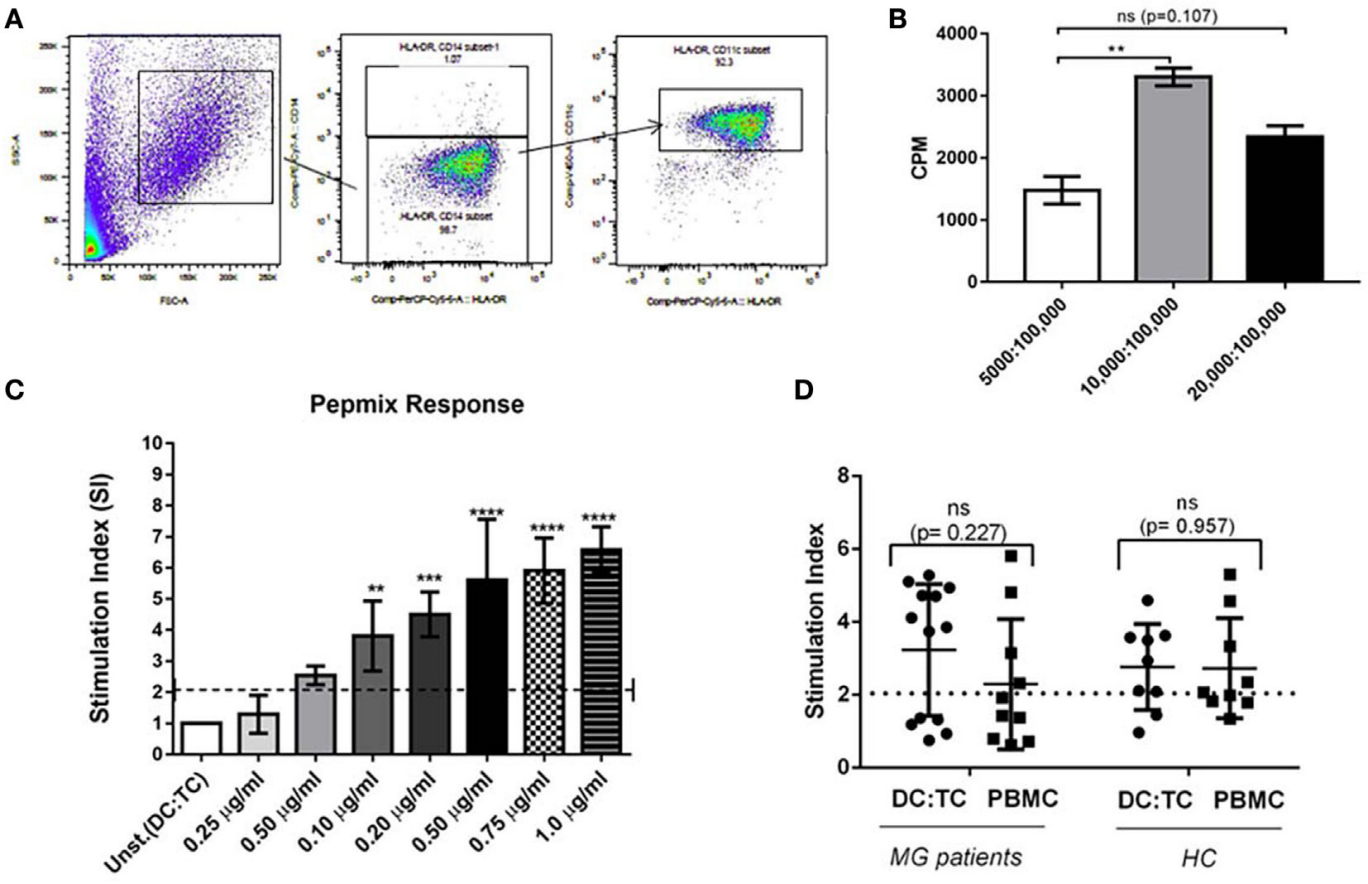
Dendritic cells (DC):TC co-cultures. Monocyte-derived dendritic cells (moDCs) were generated from healthy donors according to the protocol described in Section “Materials and Methods” in Figure 1. As a positive control CD4^+^ T cells were screened in triplicate cultures for reactivity toward a mix of control peptides, PepMix^TM^ (PM). **(A)** A representative phenotypic analysis of mDC after 5 days of culturing in GM-CSF and IL-4 using flow-cytometry. **(B)** Identifying optimal conditions for CD4^+^ T cell responses in triplicate DC: TC cultures with Pepmix^TM^ (1 μg/ml). Testing different ratios of DC:TC. Results are given as means of SI ± SD of three independent experiments with cells from a single healthy donor **(C)** CD4^+^ T cell responses against different concentrations of Pepmix^TM^ (from 0.25 µg/ml to 1.0 µg/ml) in triplicate DC:TC cultures at a 1:10 ratio. The cells were stimulated with different doses of Pepmix^TM^ for 72 h and values are given as mean SI ± SD of three independent experiments with cells from a single healthy donor. One-way analysis of variance was performed to compare the Pepmix^TM^ responses between unstimulated and Pepmix^TM^ stimulated DC:TC co-cultures. *p* values are represented by **p < 0.01, ***p < 0.001, and ****p < 0.0001. **(D)** CD4^+^ T cell responses to Pepmix^TM^ (1 ug/ml) in DC:TC or peripheral blood mononuclear cell (PBMC) cultures from myasthenia gravis (MG) patients and healthy controls (HC). For MG; DC:TC *n* = 14; PBMCs *n* = 10; for HC DC:TC *n* = 9; PBMCs *n* = 9. Values are given as individual and mean stimulation index (SI) of triplicate wells for DC:TC and PBMC cultures from MG and healthy controls (HC). Unpaired *t*-test was performed with the means comparing DC: TC and PBMCs cultures (*p* > 0.05).

DC:TC co-cultures were established for 13 MG patients and 10 HC using the moDCs and autologous CD4^+^ T cells (1 × 10^5^/well) at a ratio of 1:10. We developed an optimal protocol for generating moDCs and for co-culturing of these cells together with enriched CD4^+^ T cells, which had been frozen in liquid nitrogen from days 1 to 5 prior to co-culturing (Figure 1). We obtained cell viabilities >95% in the moDC cultures on day 5 of culturing with rhIL-4 and rhGM-CSF and significant maturation into CD11c^high^MHCII^high^ CD14^-^ moDCs had occurred (Figures 1 and 2A). Cultures were stimulated with the individual hAChR peptides (at 10 µg/ml) in triplicates in 96-well microtiter plates (Nunc) in IMDM supplemented with 10% heat inactivated FBS and incubated at 37°C in a humidified CO_2_ (5%) incubator for 72 h. For comparison of culture conditions we also isolated PBMCs from seven of the MG patients, which were cultured under the same conditions. Controls included, unstimulated cells and positive controls with PBMCs and DC:TC cultures stimulated with PepMix^TM^ (JPT Technology, Netherlands), which consisted of a mix of MG-unrelated synthetic peptides (1 µg/ml), according to the manufacturer’s description. An optimal ratio between moDC and CD4^+^ T cells was determined in the presence of different doses of PepMix^TM^ (Figure 2C). After 72 h incubation T cell proliferation was assessed by DNA-incorporation of [3H]-thymidine (PerkinElmer, Boston, MA, USA) (at 1 mCi) for 8 h. A beta-scintillation counter (Beckman Coulter, Turku, Finland) was used to measure [3H]-thymidine incorporation. The results were given as mean counts per minute (cpm) ± SD and recalculated to give the stimulation index (SI ± SD; mean cpm of stimulated/unstimulated cells). A SI value >2.0 was considered significantly enhanced (35). Culture supernatants were analyzed for cytokine content.

### Cytokine and Antibody Measurements

Anti-AChR and anti-MUSK antibodies in serum were assessed by ELISA according to the manufacturers instructions (RSR Limited, Cardiff, UK, IBL International, Hamburg, Germany). Cytokines (IFNγ, IL-17, and IL-10) were determined in supernatants from the DC:TC cultures using a Bio-plex magnetic bead based assay kit and analyzed on a Bioplex MAG-PLEX multiplex reader (Bio-rad, X,Y), according to the manufacturer’s instructions. Results are given in pg/ml ± SD and the sensitivity for detection of cytokines were 0.1–0.2 pg/ml.

### Statistical Analysis

Results are given as mean SI ± SD. Statistical significance was analyzed using multiple paired or unpaired *t*-test or one-way analysis of variance (ANOVA) with Kruskal–Wallis test using the Graph pad Prism 5.00 software (San Diego, CA, USA). *p* values are given as * < 0.05 and ** < 0.005.

## Results

### Establishing DC:TC Co-culture Conditions

Patients were recruited from the outpatient clinic at the Sahlgrenska University Hospital, Gothenburg, Sweden. Only patients with a clinical diagnosis of MG without immunosuppressive medication were included. Blood samples were drawn from individual patients and HCs after informed consent had been given. The serum was analyzed for AChR- and MUSK-specific antibodies. All MG patients were also analyzed for their HLA-DRB1, -DQA1, and -DQB1 genotypes. We observed a strong prevalence of certain haplotypes; DRB1*04/DRB1*15, DQA1*01/DQA1*03, and DQB1*03/DQB1*06 in our MG patients (Table 1). Most MG patients carried serum antibodies against AChR, but none had anti-MUSK antibodies and all HCs were negative for both types of antibodies (Table 1). The culture conditions for the DC:TC co-cultures were established in a set of pilot experiments, as described in the Section “Materials and Methods” (Figures 1 and 2A,B). Enriched CD4^+^ T cells were frozen in liquid nitrogen from days 1 to 5 prior to co-culturing with the autologous DC. Cells were stimulated with selected peptides from the AChR and PepMix^TM^, which was used as a positive control. Comparable T cell proliferation (mean SI ± S.D. 3.29 ± 1.73) to the PepMix^TM^ was observed in MG patients and HCs (mean SI ± S.D. 2.94 ± 1.18) (Figures 2C,D).

### MG Patients Respond to Multiple Peptides from the AChR

The T cell responses to AChR peptides were evaluated using the DC:TC co-culture system in 13 MG patients. CD4^+^ T cell proliferation to a panel of AChR peptides (Table 2) was assessed after 8 days of culturing, i.e., 3 days after adding the enriched CD4^+^ T cells and peptide to the cultures. The results of the DC:TC proliferation assay with CD4^+^ T cells from 13 MG patients or 10 HCs are shown in Table 3. None of the HCs responded to any of the 6 peptides while 7/10 controls responded to the PepMix^TM^ (Table 3). Of the MG patients, 11/13 exhibited CD4^+^ T cell responsiveness to the peptides with a mean SI of 3.7 (enhanced if >2-fold above background) (Table 3). Noteworthy, neither patient MG1 nor MG2 responded to the peptides, MG1 also failed to respond to the PepMix^TM^ (Table 3). For technical reasons, patients, MG6 and MG12, were not tested with peptides P1 and P3. Interestingly, there appeared to be a hierarchy between the peptides that elicited a CD4^+^ T cell response in MG patients with the most frequently recognized peptides being P2, P5, and P6. In individual MG patients, the strongest responses (SI > 4) were observed with peptides P2, P4, P5, and P6 (Table 3).

**Table 3:** Recognition of selected peptides from the hAChR by highly enriched CD4^+^ T cells isolated from peripheral blood of MG patients or HCs.

MG Patient	Age	P1	P2	P3	P4	P5	P6	PM
MG-1	28	1.41 ± 0.63	0.69 ± 0.41	1.24 ± 0.34	1.18 ± 0.52	1.07 ± 0.55	1.04 ± 0.56	1.32 ± 0.57
MG-2	33	1.31 ± 0.18	1.25 ± 0.09	0.67 ± 0.12	1.47 ± 1.08	1.3 ± 0.48	1.81 ± 0.24	4.94 ± 2.02
MG-3	33	1.99 ± 0.40	2.17 ± 0.85	2.68 ± 0.67	3.18 ± 0.75	3.25 ± 0.32	1.93 ± 1.82	4.12 ± 2.32
MG-4	36	1.89 ± 0.91	4.95 ± 1.37	2.57 ± 0.14	2.08 ± 0.96	2.48 ± 0.54	2.38 ± 0.38	3.74 ± 2.44
MG-5	50	0.78 ± 0.06	2.56 ± 1.49	0.55 ± 0.20	0.92 ± 0.11	2.15 ± 0.68	1.99 ± 1.00	0.76 ± 0.06
MG-6	52	NP	1.67 ± 0.93	NP	1.96 ± 1.38	1.65 ± 0.56	2.06 ± 0.62	1.36 ± 0.06
MG-7	55	6.34 ± 0.55	7.34 ± 1.41	6.3 ± 1.48	8.25 ± 0.53	5.14 ± 0.94	6.54 ± 0.37	4.71 ± 0.99
MG-8	60	4.12 ± 4.45	7.83 ± 4.51	0.73 ± 0.30	1.26 ± 0.79	3.95 ± 0.78	2.85 ± 1.94	5.28 ± 4.99
MG-9	64	2.67 ± 1.32	2.53 ± 1.38	2.86 ± 1.11	2.53 ± 1.06	2.52 ± 1.34	2.1 ± 0.89	5.11 ± 3.98
MG-10	74	1.4 ± 0.27	1.87 ± 0.25	1.3 ± 0.42	2.49 ± 0.39	1.92 ± 0.27	1.49 ± 0.18	4.73 ± 2.50
MG-11	82	1.31 ± 0.01	3.4 ± 2.92	1.26 ± 0.34	1.64 ± 0.57	6.11 ± 1.84	4.66 ± 5.53	1.19 ± 0.17
MG-12	86	NP	1.09 ± 0.10	NP	1.85 ± 0.09	0.95 ± 0.27	3.41 ± 0.20	0.94 ± 0.11
MG-13	86	1.22 ± 0.75	3.28 ± 3.58	3.53 ± 1.07	4.40 ± 0.91	4.72 ± 1.73	4.32 ± 1.00	3.86 ± 1.30
**Healthy control**								
HC-1	24	1.02 ± 0.16	1.18 ± 0.22	1.55 ± 0.22	0.98 ± 0.12	1.01 ± 0.26	1.25 ± 0.07	3.50 ± 0.11
HC-2	25	1.03 ± 0.24	0.14 ± 0.03	1.27 ± 0.60	0.84 ± 0.05	1.02 ± 0.43	1.05 ± 0.73	2.99 ± 1.10
HC-3	33	0.86 ± 0.22	0.66 ± 0.19	0.98 ± 0.07	0.97 ± 0.08	0.99 ± 0.20	0.88 ± 0.07	1.45 ± 0.21
HC-4	33	1.47 ± 0.54	0.88 ± 0.39	1.17 ± 0.38	1.61 ± 0.286	1.28 ± 0.54	1.24 ± 0.18	0.48 ± 0.48
HC-5	34	0.23 ± 0.20	0.12 ± 0.11	0.53 ± 0.12	0.95 ± 0.10	0.87 ± 0.18	0.98 ± 0.11	2.09 ± 1.04
HC-6	36	1.56 ± 0.56	0.89 ± 0.17	1.04 ± 0.16	1.32 ± 1.00	1.21 ± 0.53	1.10 ± 0.49	0.97 ± 0.14
HC-7	40	1.02 ± 0.55	1.72 ± 0.30	1.99 ± 2.07	1.75 ± 0.57	1.85 ± 0.85	0.79 ± 0.69	2.12 ± 0.54
HC-8	40	0.93 ± 0.16	1.08 ± 0.86	0.57 ± 0.19	0.62 ± 0.05	0.65 ± 0.04	0.35 ± 0.31	3.63 ± 0.34
HC-9	45	1.24 ± 0.59	1.62 ± 1.58	1.27 ± 0.80	1.31 ± 0.81	1.72 ± 0.73	0.76 ± 0.92	3.58 ± 0.03
HC-10	55	0.69 ± 0.33	1.87 ± 1.04	1.32 ± 0.63	1.1 ± 0.04	0.63 ± 0.04	0.68 ± 0.22	4.6 ± 3.15

*The novel DC:TC co-culture method was used. Stimulation index (SI) ± SD values in red boxes indicate responding DC:TC co-cultures from MG patients or healthy controls (HC); SI ± SD values in blue boxes indicate significant responses (SI > 2.0) to a Pepmix (PM) used as a positive DC:TC co-culture control, NP, Not performed; SI was calculated and given as means ± SD (see Materials and Methods)*.

### Comparison between the DC:TC Co-culture System and PBMC Cultures for Detection of Peptide Reactive CD4^+^ T Cells

**Figure 3:**
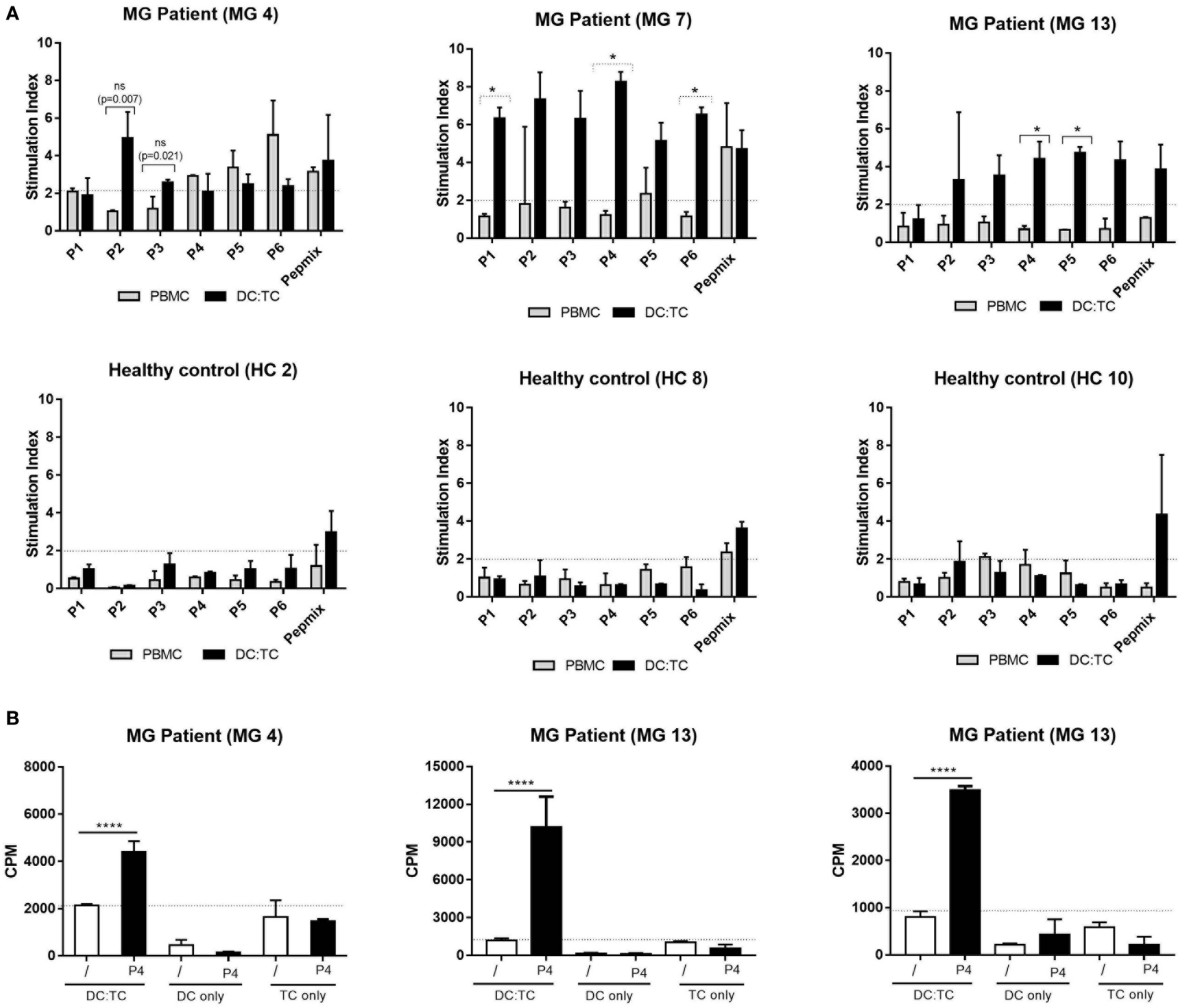
A comparison between dendritic cell (DC):TC and conventional peripheral blood mononuclear cell (PBMC) cultures for detection of acetylcholine receptor (AChR)-specific recognition. We cultured conventional PBMC (*n* = 3) or DC:TC co-cultures from myasthenia gravis (MG) patients (MG patient 4,7, and 13 in Table 1) or healthy controls (HC) (HC 2, 8, and 10) to assess reactivity against six selected hAChR peptides (*n* = 3). **(A)** Values are given as mean stimulation index ± SD of triplicate wells. Multiple *t*-test was performed to compare the peptide responses individually between the DC:TC and PBMCs groups. Medium controls of unstimulated DCs/PBMCs had the following background cpm levels: MG 4 PBMCs- 783 cpm; MG 7 PBMCs-1006; MG 13 PBMCs- 834; MG 4 DC:TC- 630; MG 7 DC:TC- 750; MG 13 DC:TC- 103; HC2 PBMCs- 677; HC8 PBMCs- 1589; HC 10 PBMCs- 1673; HC2 DC:TC- 615; HC8 DC:TC- 680; HC10 DC:TC- 770. **(B)** Values in cpm ± SD are given also for cultures from MG patient 4,7, and 13 containing DC:TC, only DCs or only T cells in medium (/) or stimulated with P4. Reactivity to the other peptides was also negligible in DC- or TC-only cultures. One-way analysis of variance was performed with *p* values represented by ****p < 0.001.

Of the 13 MG patients we selected the first 7 to be enrolled in a comparative study between DC:TC co-cultures and the conventional PBMC cultures for which was the most sensitive assay to identify reactivity to the panel of AChR-peptides. Strikingly, we found that DC:TC co-cultures were more sensitive than conventional PBMC cultures to identify reactivity to the AChR peptides (Figure 3; Table 4). As shown in Figure 3, using the DC:TC co-cultures, the three MG patients exemplified here exhibited a positive proliferative response against a majority of the peptides, while proliferation in the PBMC cultures was mostly below the detection threshold (Figures 3A,B). The difference was statistically significant. Peptide-induced responses were negative in control cultures with only T cells or only DC (Figure 3B). In fact, when summarizing the study in Table 4 it was evident that DC:TC co-cultures were superior to conventional PBMC cultures in detecting CD4^+^ T cell peptide recognition and only in one MG patient (MG4) did we detect a response that was of comparable range in the two assay systems (Table 4). However, in all other patients we observed no or very weak CD4^+^ T cell recognition of AChR-peptides in conventional PBMC cultures. By contrast, significant reactivity to several peptides was recorded in the DC:TC co-cultures (Table 4). Among the analyzed peptides, P2, 4, and 5 were found to elicit the highest recognition frequency of CD4^+^ T cells in MG patients (Table 4). Taken together, we conclude that DC:TC co-cultures were clearly superior to conventional PBMC cultures in detecting CD4^+^ T cell AChR-peptide recognition in MG patients. Importantly, no recognition of any of the peptides was found with CD4^+^ T cells isolated from HC in either the conventional PBMC cultures or in the DC:TC co-culture system, albeit reactivity to the PepMix^TM^ was comparable to that in MG patients (Table 4).

**Table 4:** A side-by-side comparison between the dendritic cell (DC):TC co-culture system and conventional peripheral blood mononuclear cell (PBMC) cultures for detection of hAChR-peptide-responsive CD4^+^ T cells in myasthenia gravis (MG) patients.

MG Patients	Culture type	Peptides					
		P1	P2	P3	P4	P5	P6
MG3	DC:TC	1.99 ± 0.40	2.17 ± 0.85	2.68 ± 0.67	3.18 ± 0.75	3.25 ± 0.32	1.93 ± 1.82
	PBMC	1.19 ± 1.40	0.76 ± 0.31	1.10 ± 1.22	1.96 ± 1.02	2.16 ± 1.02	2.12 ± 1.29
MG-4	DC:TC	1.89 ± 0.91	4.95 ± 1.37	2.57 ± 0.14	2.08 ± 0.96	2.48 ± 0.54	2.38 ± 0.38
	PBMC	2.1 ± 0.158	1.04 ± 0.05	1.18 ± 0.64	2.92 ± 0.04	3.37 ± 0.89	5.1 ± 1.83
MG-5	DC:TC	0.78 ± 0.06	2.56 ± 1.49	0.55 ± 0.20	0.92 ± 0.11	2.15 ± 0.68	1.99 ± 1.00
	PBMC	1.16 ± 0.50	0.5 ± 0.12	1.46 ± 0.90	1.51 ± 0.28	2.72 ± 0.19	1.03 ± 0.26
MG-7	DC:TC	6.34 ± 0.55	7.34 ± 1.41	6.3 ± 1.48	8.25 ± 0.53	5.14 ± 0.94	6.54 ± 0.37
	PBMC	1.13 ± 0.14	1.79 ± 0.69	1.61 ± 0.32	1.21 ± 0.22	2.34 ± 1.37	1.15 ± 0.37
MG-9	DC:TC	2.67 ± 1.32	2.53 ± 1.38	2.86 ± 1.11	2.53 ± 1.06	2.52 ± 1.34	2.10 ± 0.89
	PBMC	1.48 ± 0.32	1.97 ± 0.14	1.97 ± 0.83	1.72 ± 0.18	1.93 ± 0.91	1.53 ± 0.39
MG-10	DC:TC	1.4 ± 0.27	1.87 ± 0.25	1.3 ± 0.42	2.49 ± 0.39	1.92 ± 0.27	1.49 ± 0.18
	PBMC	1.62 ± 0.39	1.45 ± 0.26	1.74 ± 0.83	0.5 ± 0.33	0.75 ± 0.55	1.53 ± 0.14
MG-13	DC:TC	1.22 ± 0.75	3.28 ± 1.41	3.53 ± 1.07	4.4 ± 0.914	4.72 ± 3.07	4.32 ± 1.00
	PBMC	0.81 ± 0.73	0.92 ± 0.49	1.05 ± 0.32	0.69 ± 0.18	0.67 ± 0.07	0.71 ± 0.54

*Stimulation index (SI) values in red boxes indicate a significant AChR-peptide response (i.e., SI > 2.0) in DC:TC co-cultures and green boxes indicate significant responses in PBMC cultures from 7 MG patients (see Table 1); SI values were calculated as described in the Section “Materials and Methods” and given as means ± SD*.

### Cytokine Production by CD4^+^ T Cells from MG Patients

**Figure 4:**
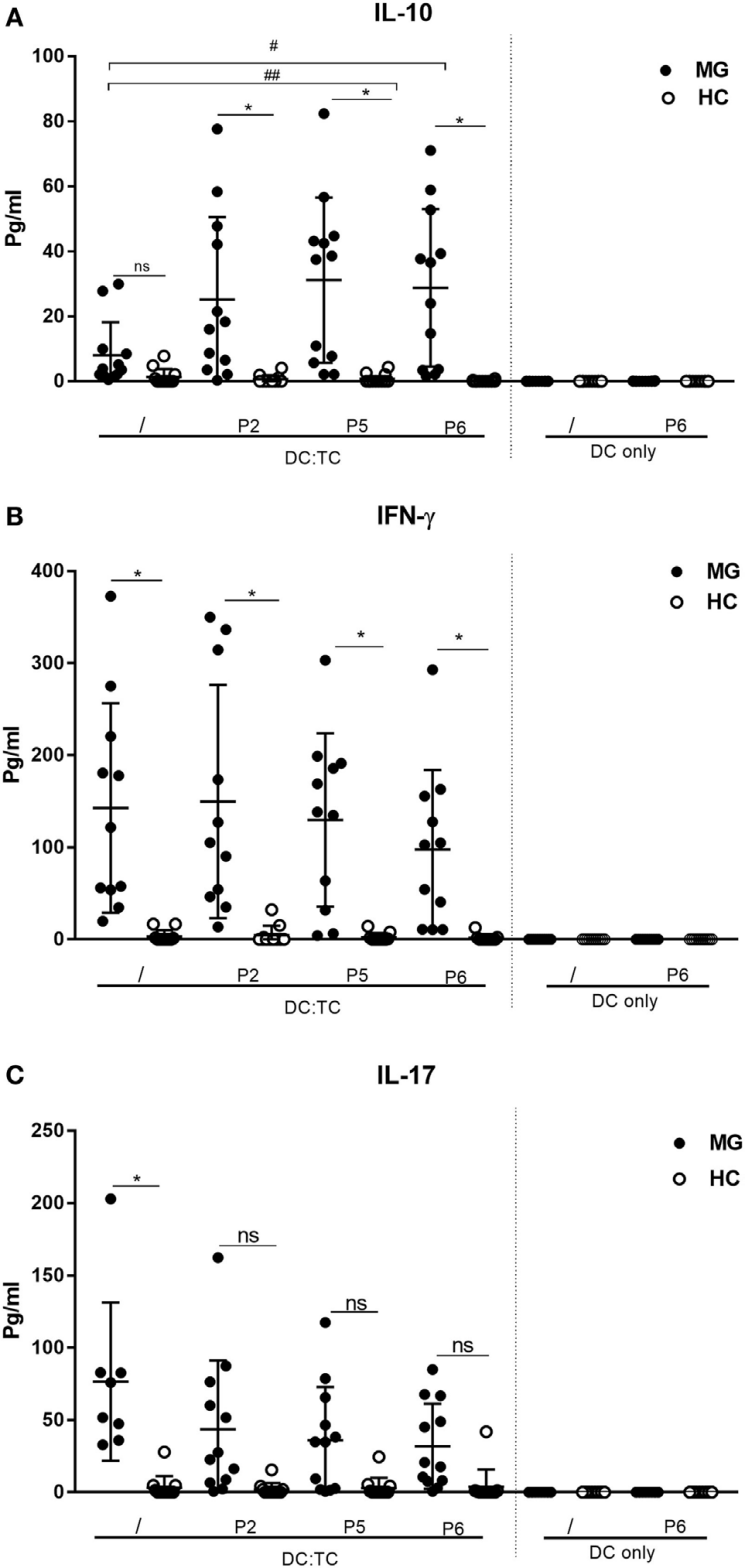
Cytokine production by dendritic cell (DC): TC co-cultures stimulated with three different hAChR peptides (see Table 2) for 72 h. Production of cytokines in pg/ml is given for DC:TC and only DC cultures. **(A)** IL-10, **(B)** IFNγ, and **(C)** IL-17. The cultures were performed in duplicates and mean production in pg/ml ± SD is shown. Note the high spontaneous production of IL-17 and IFNγ in the cultures from MG patients and the IL-10 augmenting effects by peptide (P2, P5 and P6) stimulation in DC:TC cultures. One-way analysis of variance was performed with *p* values represented by *p < 0.001 and #*p* < 0.05 between myasthenia gravis (MG) and healthy controls (HC) and between unstimulated and peptide-stimulated MG cultures, respectively.

To investigate CD4^+^ T cell subset activities in the stimulated DC:TC cultures we determined the production of IFNγ, IL-17, and IL-10 in supernatants from MG patients and the HC group against three selected peptides (P2, P5, and P6), giving the best CD4^+^ T cell proliferative responses. We found that MG patients exhibited most often elevated levels of IFNγ and IL-17 (*p* < 0.001) in cultures without recall antigen. This spontaneous production of cytokine was not observed in DC:TC cultures from HC (Figure 4). Most remarkably, though, we found peptide stimulation to increase IL-10 production in supernatants from MG patients (Figure 4). In fact, we observed significantly increased IL-10 levels relative to medium controls for P5 (#*p* < 0.05) and P6 (##*p* < 0.05) in MG patients. By contrast, for IFN-γ and IL-17 production in DC:TC cultures we did not observe significant recall responses to the peptides as opposed to the spontaneous production of these cytokines (Figure 4). Of note, the HC group did not respond with cytokine production, including IL-10, to peptide stimulation, nor did we observe peptide-induced cytokine production in DC-only cultures (Figure 4).

## Discussion

In the present study, we demonstrate that the DC:TC co-culture technique is significantly more sensitive and reliable than conventional PBMC culture techniques for detecting CD4^+^ T cell peptide recognition in MG patients. Although the method is more laborious and time consuming than the whole PBMC or CD8^+^-depleted PBMC culture methods, it allows for a higher CD4^+^ T cell density to be screened for peptide recognition, which has merits when the frequency of responsive CD4^+^ T cells is low as in MG patients (34, 35) Moreover, the antigen-presenting cell (APC) function is dependent on moDCs in DC:TC co-cultures and since DC frequencies are very low (1%) in blood the APC function relies primarily on monocytes and B cells, and not DCs, in the PBMC cultures (38). These differences, most likely, account for the higher sensitivity of the DC:TC co-cultures that we observed. We generated moDCs by culturing monocytes with GM-CSF and IL-4 for 5 days, which resulted in a high density of phenotypically distinct moDC, i.e., CD11c^high^MHCII^high^ CD14^-^ cells. MACS-purification of monocytes proved superior to plastic adherence-only techniques yielding higher purity (>95%) and twice the number of cells in culture.

In agreement with previous publications, nearly all MG patients exhibited CD4^+^ T cell reactivity to AChR-derived peptides in peripheral blood, whereas HC did not (35, 39, 40, 41). Therefore, a SI >2.0 was considered significant (35). Accordingly, when using a cutoff of >2.0 for the comparison between DC:TC co-cultures and PBMC cultures, we found that 7/7 MG patients responded with CD4^+^ T cell AChR-peptide recognition while only 4/7 patients responded using the conventional PBMC culture. Thus, in 3/7 patients the conventional PBMC cultures failed to identify responsive CD4^+^ T cells in MG patients. Furthermore, a majority (5/7) of the responding DC:TC co-cultures exhibited a positive result for at least four peptides, while this responsiveness was only found in one MG patient when using PBMC cultures. In previous reports the average response-rate to peptides in MG patients was rather variable, but clearly multiple peptides could be recognized in MG patients (28, 35, 42). This was, indeed, confirmed with DC:TC co-culture where 8/13 patients responded to two or more peptides. The three peptides that most patients reacted to were P2 (48-67), P5 (146-162), and P6 (195-212), which agrees well with observations made by others (34–36, 43).

Deitiker et al. (44) demonstrated that the P5 (146–162) peptide elicited stronger responses in patients with DQA1*0301 than in DQA1*0401 while we observed the highest SI in patients with DQA1*01 (36). Our findings confirmed the previous reports for MG patients (13, 15, 36). However, T cell proliferation was low to moderate in most published PBMC culture studies with a mean SI of 2–3, whereas with the DC:TC co-culture the response range was increased to a SI of 2–9 and 7/13 patient samples exhibited a mean SI > 3 (35). Hence, this method was not only more sensitive, but it also revealed a broader range of peptide recognition in CD4^+^ T cells from MG patients than the conventional PBMC cultures. This could be important when deciding on which peptides to include in various therapies with the aim of inducing immune tolerance in autoaggressive CD4^+^ T cell populations.

We observed a strong prevalence of the DRB1*4/DRB1*15, DQA1*01/DQA1*03, and DQB1*03/DQB1*06 haplotypes in Swedish MG patients. This HLA-restriction pattern corresponds well with what has previously been reported for MG patients with a Caucasian background (13, 15, 36). We found that MG patients with the DRB1*04/DRB1*15 haplotype recognized a majority of the six peptides. Seven of our patients had DRB1*04 and five had DRB1*15, which was recently reported to identify LOMG in Norwegian MG patients (14). This allele is known to be quite common in European and Asian populations and it is also a genetic risk factor for multiple sclerosis (45). However, it is not known whether the occurrence of LOMG is lower in populations of African origin where DRB1*15:01 is less frequent.

Current treatments for autoimmune diseases are not curative but rather control the symptoms. In MG, the first line of treatment given to most patients is symptomatic, treating the muscle fatigability by using acetylcholinesterase inhibitors (46, 47), while some patients need additional treatment with steroids or other forms of immunosuppressants (46, 47). Because the disease is gradual and progressive most patients require long-term medication (7). A prerequisite for high-affinity anti-AChR autoantibodies is the presence of auto aggressive AChR-specific CD4^+^ T cells (23). Both Th1 and Th17 CD4 T cells have been implicated in MG disease and enhanced production of IFNγ and IL-17, similar to our findings, has been documented in earlier studies (19, 48–50). Therefore, there is growing interest in developing novel therapies, which may suppress auto aggressive Th1 and Th17 cells and reinstate tolerance in the immune system (38). If successful, such immune tolerance inducing therapies will completely change the way autoimmune patients are treated since tissue destructive CD4^+^ T cells may be suppressed or eliminated, hence halting the disease driving mechanisms. Accordingly, new treatments to induce tolerance in MG could be a way forward to prevent progression and possibly offer cure from disease (3). Indeed, several publications suggest that restoring a functional Treg population could be a curative therapeutic intervention in MG (51–53). One such approach has focused on identifying the immunodominant epitopes in the AChR to use these for protective immunizations (3, 54). Several studies have described dominant antigenic epitopes in AChR, which could be candidates and used to vaccinate MG patients (54–57). Indeed, recent findings suggest that restoring a functional Treg population using immunodominant epitopes from the AChR could be an effective therapeutic approach for MG disease (5, 53, 58).

Interestingly, the most striking effect of AChR-peptide stimulation of CD4^+^ T cells from MG patients in our study was the induction of IL-10 production in DC:TC co-cultures. This cytokine is primarily associated with Treg functions and immunosuppression (59). Treatment with dominant AChR-peptides would, therefore, be a way to stimulate these IL-10 producing, potentially Tregs and reinstate tolerance in MG patients. Surprisingly few reports have documented cytokine production from CD4^+^ T cells in MG patients, let alone studies, including IL-10 production (48–50, 60–64). However, several studies have demonstrated that AChR-specific IL-10 production by PBMCs, was increased by immunosuppressive drug treatment, while a concomitant reduction was clearly observed for IL-17 and IFNγ-production (50, 65, 66). Hence, our observation that IL-10 production was elicited from peptide-stimulated circulating MG-specific CD4^+^ T cells further supports the notion that re-instating tolerance through stimulation of Tregs could be a way forward for treatment of MG disease.

Given the extended arsenal of very sensitive detection methods for the presence of single antigen-specific CD4^+^ T cells in *in vitro* cultures we anticipate that the sensitivity of the DC:TC co-culture system could be even more improved. In particular, adding ELISPOT, intracellular cytokine or tetramer detection assays rather than proliferation assays or cytokines in supernatants, as used here. Nevertheless, the present study provides clear evidence for the importance of selecting a sensitive assay when analyzing frequencies of reactive CD4^+^ T cells in peripheral blood from MG patients. We found that DC:TC co-cultures were much superior to traditional PBMC cultures for detection of AChR-peptide recognition. It also identified the presence of potentially, AChR-peptide reactive, IL-10 producing Tregs in the peripheral blood of MG patients.

## Ethics Statement

The study was approved by the regional ethical review board at the University of Gothenburg, Sweden.

## Author Contributions

NL, SS, and MV contributed to the design of the study and for planning the experiments and SS and NL wrote the manuscript. CL and CM were responsible for the ethical permission and for the clinical selection and diagnostic evaluation of patients and controls. SS, MV, KS, and SM performed the experiments. SS organized figures and tables.

## Conflict of Interest Statement

NL has intellectual property related to MG treatment. NL and MV are affiliated with the Toleranzia AB company that develops MG-specific treatments based on fusion proteins. Disclosures are managed in compliance with the policies of the University of Gothenburg, Sweden. SS, CM, CL, SM, and KS have nothing to disclose.
